# “Here we go again”: the inspection of collagen extraction protocols for ^14^C dating and palaeodietary analysis

**DOI:** 10.1080/20548923.2021.1944479

**Published:** 2021-07-20

**Authors:** Sahra Talamo, Helen Fewlass, Raquel Maria, Klervia Jaouen

**Affiliations:** a Department of Human Evolution, Max Planck Institute for Evolutionary Anthropology, Leipzig, Germany; b Department of Chemistry G. Ciamician, Alma Mater Studiorum, University of Bologna, Bologna, Italy; c Ilse Katz Institute for Nanoscale Science & Technology, Ben Gurion University of the Negev, Beer-Sheva, Israel; d Géosciences Environnement Toulouse, Observatoire Midi Pyrénées, Toulouse, France

**Keywords:** Collagen, ultrafiltration, radiocarbon dating, palaeodiet, FTIR, bone pretreatment

## Abstract

Archaeological bone collagen is highly useful for radiocarbon (^14^C) dating and palaeodietary reconstruction. However, collagen preservation and carbon contamination are essential considerations when extracting collagen, becoming especially crucial close to the limit of the method (50,000 years before present = BP). Strong progress has been achieved in the past two decades by ^14^C and stable isotopic laboratories in removing contamination from archaeological bones, but different pretreatment protocols have been proven to produce varying results. Here we compare three collagen extraction protocols used for palaeodietary studies and ^14^C dating, considering collagen yield, elemental and stable isotopic data, FTIR analysis, and ^14^C dates. We focus on the impact of ultrafiltration on the yield and quality of the extracted material. The results again underline the importance of rigorous decontamination methods to gain accurate ^14^C dates and demonstrate that different protocols have significant effects on the quality and yield of extracted collagen.

## 1. Introduction

Collagen extracted from archaeological bones and teeth is one of the most important biomolecules for radiocarbon (**^14^**C) dating and palaeodietary studies. Collagen Type I comprises ∼90% of the organic portion of the mammalian bone (∼22% dry weight of bone). The molecule is comprised of three peptide chains organised in a triple helix structure with a molecular weight of ∼285-300** **kDa (∼90-100** **kDa for each a peptide chain) (Furthmayr and Timpl 1971; Collins et al. 2002; Zhang, Li, and Shi 2006; Garnero 2015). A key concern of laboratories specialising in ^14^C dating or palaeodietary analysis of archaeological bone is the refinement of methods to extract and purify collagen for analysis. This is hampered by three key issues:

**1)** The degradation of collagen through the rapid or gradual breakup of the peptide chains. This is strongly influenced by environmental conditions, with tropical or arid environments particularly detrimental to the preservation of proteins. The attack of fungi and bacteria can further alter the triple-helix sequence of mammalian collagen (Collins et al. 2002; Yu et al. 2014). In addition to hampering efforts to extract sufficient collagen for analysis, degradation can cause isotopic fractionation and therefore bias the interpretation of δ^13^C and δ^15^N values for dietary reconstruction (Masters 1987; Dobberstein et al. 2009).
**2)** Contamination with exogenous carbonaceous contaminants can affect stable isotopic values and alter ^14^C dates if the contaminant is a different age to the sample (from modern to fossil carbon). Contaminants may derive from the burial environment (such as humic acids or bacteria from the soil), during post-excavation handling and storage (including the application of conservatives) or the laboratory pretreatment and measurement (Nielsen-Marsh and Hedges 2000; Higham 2011).
**3)** Although less detrimental to ^14^C dating efforts, endogenous material (such as bone lipids) which are not removed from collagen extracts can significantly alter stable isotopic values and affect palaeodietary interpretations (Liden, Takahashi, and Nelson 1995; Jørkov, Heinemeier, and Lynnerup 2007).

To correctly determine the age of any archaeological bone sample, these issues need to be carefully considered. The age of any exogenous carbon contaminants matters. The addition of 1% fossil carbon (e.g. from organic solvents of petrochemical origin) will make ^14^C ages older by 80 years across the ^14^C timescale. Fossil contamination is, therefore, less problematic for Palaeolithic samples where 80 years is usually less than the standard error associated with the measurement. However, for Neolithic or younger samples, the offset exceeds the typical radiocarbon error for this time range of 25–40 years.

**Figure 1.**
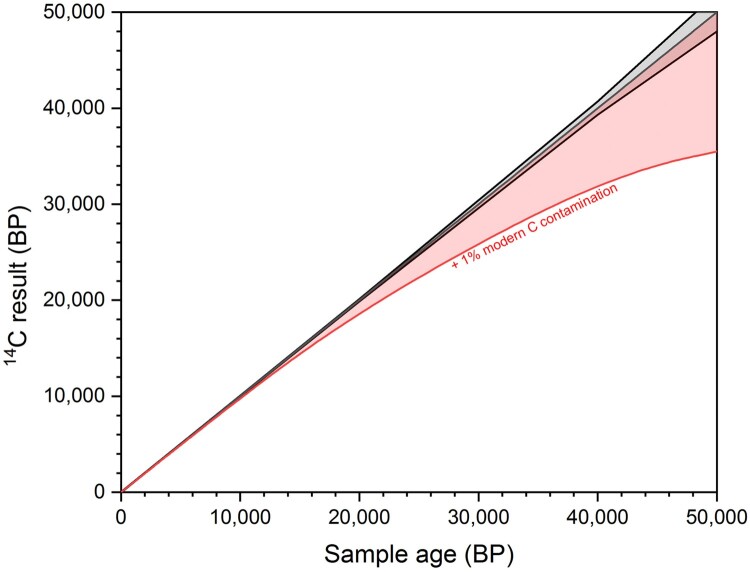
The increasing effect of modern carbon contamination with age. The black line shows a ^14^C date ± 1 SD error and the red line show the effect of adding 1% modern carbon contamination.

In contrast, the addition of modern carbon will make ^14^C ages younger, with the effects becoming increasingly catastrophic with rising age due to the exponential decay of ^14^C. For example, in a 42,000 year old bone sample, the addition of 1% of modern carbon will result in an 8,000 year shift to a younger age (Figure 1). For these reasons, when considering ^14^C dates from Palaeolithic bones, older ages are generally viewed as more likely to be accurate (Higham 2011). Due to the high risk of producing erroneous ^14^C results, modern carbon contamination should therefore be kept below 0.1% (Bronk Ramsey 2008).

Most ^14^C labs employ a collagen extraction method based on the method developed by Longin (1971). This involves an “acid–base-acid” (ABA) or “acid-alkali-acid” (AAA) sequence, with an initial step to dissolve the bone mineral in weak acid (usually HCl), followed by treatment with a weak base (NaOH) to remove humic acid contaminants, and gelatinisation in acidic water to unravel the collagen triple helix. Although the resulting gelatin solution is often called collagen, in fact, it is a mixture of soluble collagen and non-collagenous proteins (DeNiro 1985; Ambrose 1990; van Klinken 1999; Wadsworth and Buckley 2018). The addition of an ultrafiltration step (UF) following gelatinisation, first suggested by Brown et al. (1988) and more widely applied in the past two decades (Bronk Ramsey et al. 2004; Brock, Bronk Ramsey, and Higham 2007; Beaumont et al. 2010; Talamo and Richards 2011), has been an important development in purifying collagen extracts. Ultrafiltration separates the gelatin based on molecular weight, usually with a molecular weight cut-off (MWCO) around 30** **kDa (although this is not a hard boundary). The <30** **kDa fraction is thought to contain small bacterial contaminants, salts and degraded proteins whereas the >30** **kDa fraction should retain large intact collagen molecules (Brown et al. 1988; Bronk Ramsey et al. 2004; Brock et al. 2013). Ultrafiltration will therefore also retain, and even concentrate, large molecular weight contaminants, including humic acids (Szpak, Krippner, and Richards 2017a) and lipid contaminants (Guiry, Szpak, and Richards 2016) which may alter stable isotopic values and ^14^C dates. For this reason, it is important to incorporate the NaOH step to remove humic acids contaminants prior to ultrafiltration. It has been demonstrated that the ultrafilter membrane needs to be thoroughly washed prior to use to remove the humectant coating on the filter itself to avoid contamination (Bronk Ramsey et al. 2004; Higham, Jacobi, and Bronk Ramsey 2006; Brock, Bronk Ramsey, and Higham 2007; Beaumont et al. 2010; Fülöp et al. 2013). Particularly for Pleistocene samples, in many cases, the addition of this step has resulted in older ages (usually deemed to be more accurate) compared to non-ultrafiltered extracts (Hajdas et al. 2009; Higham 2011). Ultrafiltration has been adopted by many, but not all, radiocarbon laboratories (Hüls, Grootes, and Nadeau 2009; Hüls et al. 2017) due to the higher expense in lab consumables and time investment, the decrease in collagen yield through the removal of degraded but endogenous molecules, as well as concerns over contamination from the filter membrane itself (Hüls, Grootes, and Nadeau 2009; Fülöp et al. 2013).

Prior to attempting a costly radiocarbon date, the quality of the collagen extract is a crucial consideration. It is generally considered that a collagen yield of 1% weight of the original bone sample is the lowest suitable limit for obtaining reliable ^14^C dates (Hedges and van Klinken 1992; Brock, Bronk Ramsey, and Higham 2007; Dobberstein et al. 2009). Chemical indicators including the collagen yield, %N, %C, and C:N are commonly used to check if contamination and/or degradation have significantly altered the extracted collagen (DeNiro 1985; Schoeninger et al. 1989; Ambrose 1990; van Klinken 1999; Hedges 2002; Strydonck, Boudin, and Ervynck 2004; Harbeck and Grupe 2009). If the collagen is well preserved, the C:N ratio should fall between 2.9 and 3.6 (Ambrose 1990; van Klinken 1999). Collagen samples with C:N ratios falling outside of this range are considered unsuitable for dating. In general, degraded collagen samples have variable (low) %C and variable (high) C:N ratios and contaminated collagen generally has higher %C and C:N values (van Klinken 1999). Although these ranges are useful quality indicators, they are not infallible and low levels of contamination may be present in an extract without causing the values to fall outside of accepted ranges so a range of quality indicators should be considered (Schoeninger et al. 1989; Ambrose 1990; van Klinken 1999).

In addition to being crucial tools in palaeodietary analysis, δ^13^C and δ^15^N values are also helpful quality criteria when the species from which collagen was extracted is known (van Klinken 1999). Nitrogen isotopes are tracers of trophic level, while carbon isotopes can distinguish diets from marine or terrestrial environments, and if the subsistence relies on C_4_ or C_3_ plants (Schoeninger, DeNiro, and Tauber 1983). The use of these tracers in archaeological contexts reveals differences in subsistence strategies, such as highlighting the more variable diets of *Homo sapiens* compared to Neanderthals (Richards and Trinkaus 2009), the onset of agriculture in the Americas (Tykot, Burger, and Van der merwe 2006; Kennett et al. 2020), the age of weaning in ancient populations (Fuller et al. 2006) and the abrupt changes in marine food consumption at the onset of the Neolithic (Richards, Schulting, and Hedges 2003). Collagen extracts with C:N ratios falling outside the biogenic range are also discarded for dietary interpretation, due to 1) isotopic fractionation due to the loss of amino acids and protein hydrolysis (Bada, Schoeninger, and Schimmelmann 1989; Ambrose 1990; Grupe, Balzer, and Turban-just 2002) and 2) the potential for contaminants altering the stable isotopic values (Sealy et al. 2014), and thus influencing the dietary signal.

In recent decades, Fourier Transform Infrared Spectroscopy (FTIR) has also proven highly useful for characterising the quality of extracted collagen (DeNiro and Weiner 1988; Yizhaq et al. 2005; D'Elia et al. 2007) by giving information of specific bands such as Amide I, II, and III; the first results from peptide bond C=O stretch, the second results from mixed C–N stretch and N–H in-plane bend, and the third also results from mixed C–N stretch and N–H in-plane bend with additional contributions from C–Cα stretch.

As it has been extensively documented that different collagen extraction techniques yield variable results in terms of collagen yield and quality (Chisholm et al. 1983; Collins and Galley 1998; Jørkov, Heinemeier, and Lynnerup 2007; Beaumont et al. 2010; Talamo and Richards 2011; Brock et al. 2013; Fülöp et al. 2013; Sealy et al. 2014; Cersoy et al. 2017; Szpak, Krippner, and Richards 2017a), here we scrutinise three different collagen extraction protocols on a range of archaeological bones dating from >49,000 - 300 ^14^C years BP. These analyses allow us to discuss the implications of the ultrafiltration step, the importance of the collagen yield, %N, %C, C:N and stable isotopic values (δ^13^C and δ^15^N) to determine the quality of the collagen extract, and evaluate the most effective method for ^14^C dating bones and palaeodietary analysis.

## 2. Material & methods

**Figure 2.**
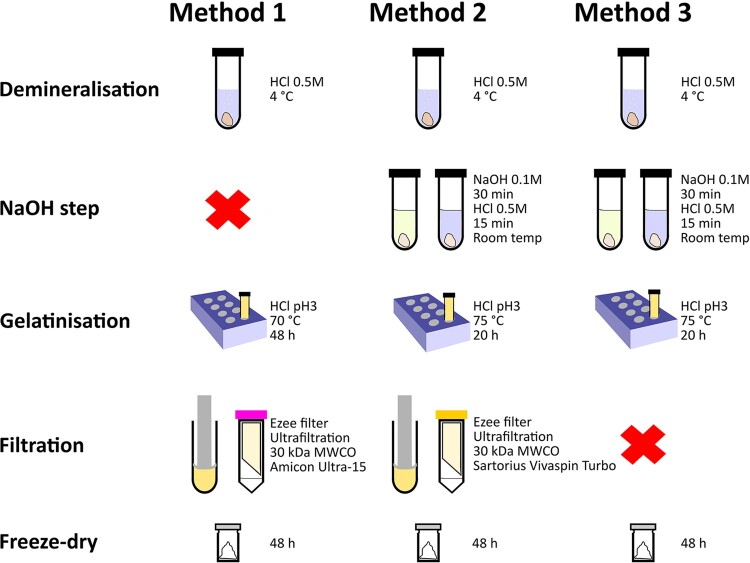
Comparison of the collagen extraction protocols used in this study. The red cross indicates exclusion of a step.

We selected a range of bones from different environments of varying age, predominantly >20,000 BP (Table 1). All bone samples were pretreated in the Department of Human Evolution at the Max Planck Institute for Evolutionary Anthropology (MPI-EVA), Leipzig, Germany following three collagen extraction protocols. Details are given below and shown in Figure 2. Two tests (Experiment A and Experiment B) were conducted to compare the different collagen extraction methods.

**Table 1.** Information of samples used in Experiments A and B.

MPI sample code	Species/Element	Geographical origin	References	Experiment	Sample num in Figures 8–11
R-EVA 123	Mammoth rib	Brown Bank, North Sea Plains	(Talamo and Richards 2011; Fewlass et al. 2018; 2019; Korlević, Talamo, and Meyer 2018)	A	
R-EVA 124	Woolly rhino long bone	Brown Bank, North Sea Plains	(Talamo and Richards 2011; Fewlass et al. 2018; 2019; Korlević, Talamo, and Meyer 2018)	A	
R-EVA 616	Horse	Sachsen-Anhalt, Germany	(Döhle 2008)	B	1
R-EVA 2393	Fauna mandible	Spain	(Micó et al. 2020)	B	2
R-EVA 2387	n/a	Aragon-Spain	This paper	B	3
R-EVA 2254	Tibia, Cervidae	Catalunia-Spain	This paper	B	4
R-EVA 2388	n/a	Aragon-Spain	This paper	B	5
R-EVA 2370	n/a	Aragon-Spain	This paper	B	6
R-EVA 557	Fauna large humerus	Catalunia-Spain	(Talamo et al., 2016a)	B	7
R-EVA 1916	Fauna long bone	Serbia	(Dogandzic et al. 2017)	B	8
R-EVA 2900	Mammalia *indet.*	Poland	(Krajcarz et al. 2014)	B	9
R-EVA 2897	Mammalia *indet.*	Poland	(Krajcarz et al. 2014)	B	10
R-EVA 2166	Helasmoterium	Russia	(Reimer and Svyatko 2016; Shpansky, Aliyassova, and Ilyina 2016); new date in this paper	B	11
R-EVA 1753	*Ursus speleo*	Herdengelhöhle-Austria	(Döppes et al. 2019)	B	12
R-EVA 1755	*Ursus speleo*	Herdengelhöhle-Austria	(Döppes et al. 2019)	B	13

### 2.1. Method 1 (palaeodietary analysis)

The bone sample is decalcified in HCl 0.5M at 4°C until no CO_2_ effervescence is observed (several hours for powder samples or days/weeks for whole bone pieces with HCl changed once per week). The resulting solid is gelatinised in a heater block at 70 °C for 48 h at pH 3. The resulting gelatin is then filtered with an Eeze-Filter™ (Elkay Laboratory Products (UK) Ltd.) to remove small (>80 µm) particles. Prior to use, the filter is sonicated for 20 min in Milli-Q water (Brock, Bronk Ramsey, and Higham 2007). The gelatin is then ultrafiltered (Amicon Ultra-15 with a Regenerated Cellulose Membrane) with a 30 kDa MWCO. Prior to use, the ultrafilters are cleaned by centrifuging once with NaOH 0.5M followed by three centrifuges with Milli-Q water, each for 20 min (based on Sealy et al. 2014). The collagen extracts were frozen for 24 h and lyophilised for 48 h. After freeze-drying, all extracts were immediately weighed on a microbalance to determine the collagen yield (%).

### 2.2. Method 2 (^14^C dating)

Bone samples are decalcified in 0.5M HCl at 4 °C for several hours (powder) until no CO_2_ effervescence is observed or for several days/weeks (whole bone) until CO_2_ effervescence has stopped and the sample is soft/translucent (HCl changed once or twice a week). 0.1M NaOH is added for 30 min to remove humic acids, followed by 0.5M HCl for 15 min. The resulting solid is gelatinised in HCl pH 3 in a heater block at 75 °C for 20 h. The gelatin is then filtered with an Eeze-Filter™ (Elkay Laboratory Products (UK) Ltd.) to remove small (>80 µm) particles (cleaned as above). The gelatin is then ultrafiltered (Sartorius Vivaspin Turbo 15 with a Polyethersulfone Membrane and 30 kDa MWCO). Prior to use, the filters are centrifuged twice for 10 min with Milli-Q water, followed by 1 h submerged in Milli-Q water in an ultrasonic bath, followed by three time 10 min rinses with Milli-Q water in the centrifuge (Bronk Ramsey et al. 2004; Brock, Bronk Ramsey, and Higham 2007). The >30 kDa and <30 kDa fractions were frozen for 24 h and then lyophilised for 48 h, and weighed immediately on a microbalance to determine the % yield.

### 2.3. Method 3 (without ultrafiltration)

The outer surface of the bone sample was cleaned by a sandblaster and then ca. 500 mg of whole bone was sampled. The demineralisation, NaOH/HCl and gelatinisation steps are identical to Method 2 above: decalcification in 0.5M HCl at fridge temperature until no CO_2_ effervescence is observed; 0.1M NaOH added for 30 min to remove humics; 0.5M HCl for 15 min; gelatinisation in HCl pH3 in a heater block at 75 °C for 20 h. After gelatinisation, the samples were centrifuged to separate particles still present before being frozen and lyophilised for 48 h. Following freeze-drying all extracts were immediately weighed on a microbalance to determine the collagen yield (%).

### 2.4. Experiment A

Experiment A compares two collagen extraction methods that have both been employed in the Department of Human Evolution at the MPI-EVA. The first (Method 1) has been in use since 2004 for collagen extraction for stable isotopic studies of palaeodiet (e.g. Richards and Schmitz 2008; Mannino et al. 2011; Britton et al. 2012; Salazar-garcía et al. 2014). Initially, the protocol followed Richards and Hedges (1999) but was updated to include an additional step to purify the collagen with Ezee-filters and ultrafiltration (Sealy et al. 2014). The second protocol (Method 2) has been in use since 2011 (Talamo and Richards 2011; Fewlass et al. 2019) for the extraction of collagen specifically for radiocarbon dating Palaeolithic bone (e.g. Hublin et al. 2012; Talamo et al. 2012; Talamo et al., 2016a; Fewlass et al. 2020; Talamo et al. 2020). Methods 1 and 2 are both modified versions of the Longin (1971) protocol with the addition of ultrafiltration, but vary in the strength, temperature and duration of the different steps.

For Experiment A, we used two samples: one mammoth bone (R-EVA 123) and one woolly rhino bone (R-EVA 124), both from the North Sea plain (Table 1). These samples have been widely used in methodological tests at the MPI-EVA (Talamo and Richards 2011; Fewlass et al. 2018; Korlević, Talamo, and Meyer 2018; Fewlass et al. 2019). For each bone, the outer surface was first cleaned by a shot blaster to eliminate impurities from the surface. Generally, Method 1 is performed on powdered bone, whereas Method 2 uses whole bone pieces, although this can vary depending on what is available. Therefore, for each bone, three aliquots of ca. 500 mg of bone powder and a whole piece of bone (ca. 500 mg) was sampled for each method so that each bone was extracted four times with each method.

### 2.5. Experiment B

In order to assess the effects of ultrafiltration on ^14^C dates, Experiment B compares Method 2 with an identical protocol that excludes the ultrafiltration step (e.g. a modified version of Longin (1971), called Method 3). In this experiment, the ^14^C results were obtained from both the >30 kDa and <30 kDa fractions of collagen extracted using ultrafiltration, as well as collagen extracted from the same bones with the omission of the ultrafiltration step (Method 3). All collagen extracts were assessed based on their collagen yield, elemental and stable isotopic values and were analysed with FTIR.

For Experiment B, we included 13 archaeological bone samples spanning different time-periods and environments (Table 1 and Supplementary Table S2). These bones had all been previously pretreated and were selected as they were known to produce a significant amount of material in the <30 kDa fraction following ultrafiltration. All 13 samples we pretreated once with our standard ^14^C dating collagen extraction protocol (Method 2) with ultrafiltration and once without ultrafiltration (Method 3).

### 2.6. Elemental and stable isotopic analysis

To assess the quality of each extract, collagen (ca. 0.5 mg) was weighed into a tin cup and analysed on a ThermoFinnigan Flash elemental analyser coupled to a Thermo Delta plus XP isotope ratio mass spectrometer (EA-IRMS). Stable carbon isotope ratios were expressed relative to VPDB (Vienna PeeDee Belemnite), and stable nitrogen isotope ratios were measured relative to AIR (atmospheric N_2_), using the delta notation (δ) in parts per thousand (‰). Repeated analysis of both internal and international standards indicates an analytical error of 0.1 and 0.2‰ (1σ) for δ^13^C and δ^15^N respectively, as well as isotope ratios in agreement with known values. Additional information is given in the Supplementary Information and Supplementary Table S3.

### 2.7. FTIR analysis

For the collagen extracts from Experiment B, ca. 0.3 mg collagen was homogenised and mixed with ∼40 mg of IR grade KBr powder in an agate mortar and pestle, pressed into a pellet using a manual hydraulic press (Wasserman) and analysed with an Agilent Technologies Cary FTIR Spectrometer with a DTGS detector. Spectra were recorded in transmission mode at 4 cm^−1^ resolution with averaging of 34 scans between 4000 and 400 cm^−1^ using Resolution Pro software (Agilent Technologies). The spectra were analysed and compared to library spectra of well-preserved collagen and bone.

### 2.8. AMS dating

For both experiments, the collagen extracts were sent to the Curt-Engelhorn-Centre for Archaeometry Klaus-Tschira-AMS facility in Mannheim, Germany (lab code: MAMS), where they were graphitised and dated using a MICADAS accelerator mass spectrometer (AMS) (Kromer et al. 2013). The samples were pretreated at roughly the same time (see Supplementary Tables S1 and S2 for details) and were measured in the same magazine in the AMS to ensure that any differences in outcome were due to the methods used rather than laboratory/instrumental background variation. Background bone samples (>50,000 BP) were pretreated and measured alongside all the samples to monitor lab-based contaminants (Döppes et al. 2019).

## 3. Results & Discussion

### 3.1. Experiment A

#### 3.1.1 Collagen yield

**Figure 3.**
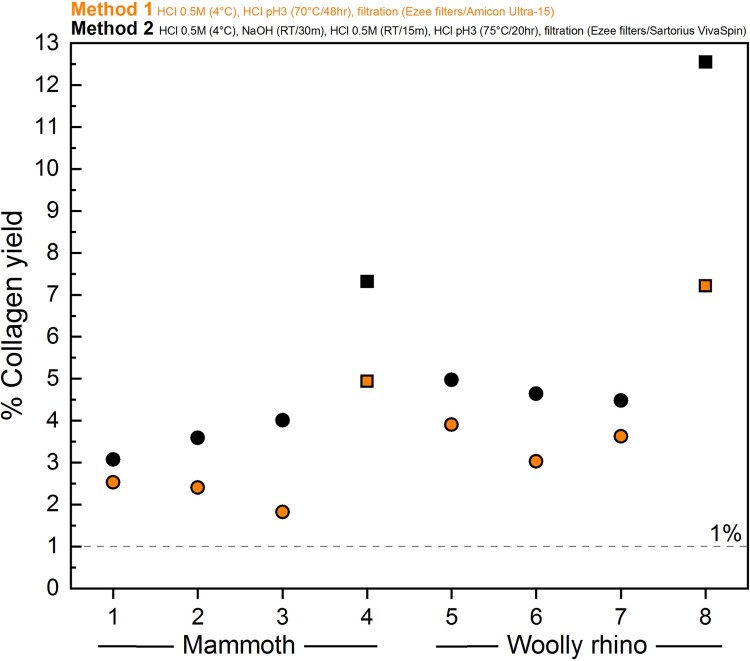
Differences in % collagen yield between the Method 1 (orange) and Method 2 (black) pretreatment protocols for the mammoth bone (samples 1-4) and woolly rhino bone (samples 5-8). Squares are whole pieces of bone and circles are bone powder samples.

The amount of collagen retrieved was lower using Method 1 compared to Method 2 (Figure 3, details in Supplementary Table S1), for both powdered bone (*mammoth* Method 1 = 2.3 ± 0.4% 1SD (n=3); *mammoth* Method 2 = 3.6 ± 0.5% 1SD (n=3); *woolly rhino* Method 1 = 3.5 ± 0.5% 1SD (n=3); *woolly rhino* Method 2 = 4.7 ± 0.3% 1SD (n=3)) and whole bone pieces (*mammoth* Method 1 = 4.9%; *mammoth* Method 2 = 7.2%; *woolly rhino* Method 1 = 7.2%; *woolly rhino* Method 2 = 12.6%). This confirms a trend reported in Colleter et al. (2019) where collagen extracted from skeletons from the Couvent des Jacobins had higher collagen yields on average when extracted with the Method 2 protocol (19 bones, mean collagen yield 8%) compared to the Method 1 protocol (99 bones, mean collagen yield 5%).

The differences in collagen yield may be related to several factors, which differ between the two methods:

1. Inclusion of the NaOH/HCl step in Method 2
2. Difference in duration and temperature of gelatinisation stage (heater block)
3. Different brands/cleaning protocols of ultrafilters between the methods

It has been noted in several studies that the NaOH step can lead to collagen loss (Chisholm et al. 1983; Liden, Takahashi, and Nelson 1995; Szpak, Krippner, and Richards 2017a), particularly in the case of poorly preserved samples. In this test, the addition of the NaOH step in Method 2 did not cause a decrease in the yield of collagen compared to Method 1, although in general, we consider these bones to be well-preserved for their age.

We observed a relatively high amount of fluffy white material (collagen) in the <30 kDa fraction of the Method 1 extracts (elemental and stable isotopic data shown in Table S1) whereas only white marks were present in the tubes of the <30 kDa fraction of the Method 2 extracts, so it appears more collagen is passing through the filter in Method 1. It is possible that this is related to the different brands of ultrafilters used (Method 1 uses Amicon Ultra-15; Method 2 uses Sartorius Vivaspin Turbo 15) as observed by Hüls et al. (2009), but both filters have a MWCO of 30 kDa. We consider the longer duration of the gelatinisation stage in Method 1 (Method 1: 70 °C, 48 h; Method 2: 75 °C, 20 h) as the most likely cause for the lower >30 kDa collagen yields obtained with Method 1, with solubilised collagen degraded by prolonged temperature and acidity (Semal and Orban 1995; Beaumont et al. 2010; Fewlass et al. 2019).

It has also been noted previously that the pretreatment of powdered or ground bone results in lower collagen yields compared to whole bone fragments (Schoeninger et al. 1989; Collins and Galley 1998; Fewlass et al. 2019). The results of this study again demonstrate this difference, with the extraction of collagen from whole pieces of bone (median 7.2%) resulting in a significantly higher yield compared to powdered bone (median 3.6%) (Kruskal–Wallis test: chi square: 7.7794, df=1, *p*-value: 0.005). The lower yield from powdered bone could be the result of damage to the collagen during drilling of the powder, an increased likelihood of sample loss during the pretreatment of powder during the multiple solvent/rinsing steps and/or the much faster demineralisation stage for powdered samples (a matter of hours) compared to pieces (slow demineralisation over days/weeks).

#### 3.1.2. Elemental and stable isotopic values

**Figure 4.**
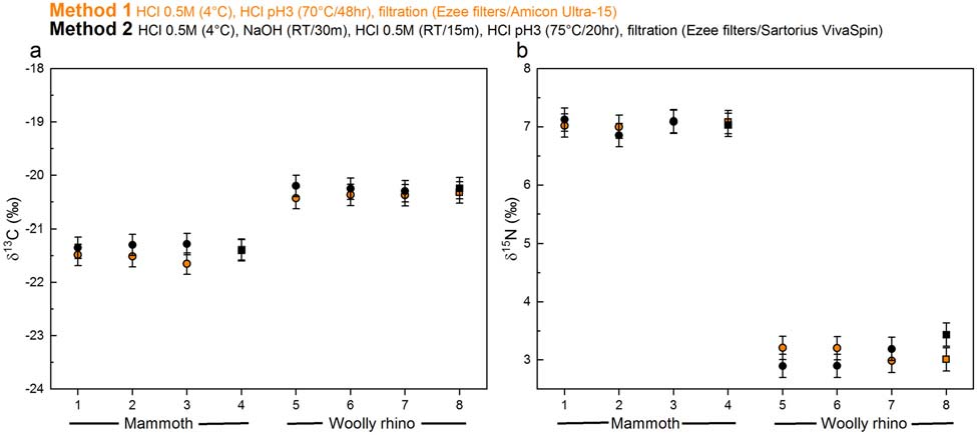
Comparison of the δ^13^C and δ ^15^N values of collagen extracted using Method 1 (orange) and Method 2 (black) from the mammoth bone (samples 1-4) and woolly rhino bone (samples 5-8). Squares are whole bone samples, and circles are bone powder samples. The error bars show an analytical error of 0.2‰ (1σ) based on repeated analysis of both internal and international standards.

The stable isotopic values obtained were in keeping with species dietary expectations. The isotopic values (δ^13^C and δ^15^N) were virtually identical between the different methods (Figure 4, Supplementary Table S1), with any differences within the measurement precision of 0.2‰ (*mammoth* Method 1 δ^13^C = −21.5 ± 0.1‰ 1SD (n=4); *mammoth* Method 2 δ^13^C = −21.3 ± 0.05‰ 1SD (n=4); *mammoth* Method 1 δ^15^N = 7 ± 0.04‰ 1SD (n=4); *mammoth* Method 2 δ^15^N = 7 ± 0.12‰ 1SD (n=4); *woolly rhino* Method 1 δ^13^C = −20.4 ± 0.04% 1SD (n=4); *woolly rhino* Method 2 δ^13^C = −20.2 ± 0.04% 1SD (n=4); *woolly rhino* Method 1 δ^15^N = 3.1 ± 0.12% 1SD (n=4); *woolly rhino* Method 2 δ^15^N = 3.1 ± 0.26% 1SD (n=4)). Likewise, the stable isotopic values of the <30 kDa fraction of the Method 1 extracts are the same as the >30 kDa fraction within instrumental error (*mammoth* Method 1 <30 kDa δ^13^C = −21.5 ± 0.06‰ 1SD (n=3); *mammoth* Method 1 <30 kDa δ^15^N = 7 ± 0.02‰ 1SD (n=3); *woolly rhino* Method 1 <30 kDa δ^13^C = −20.5 ± 0.1% 1SD (n=4); *woolly rhino* Method 1 <30 kDa δ^15^N = 3.1 ± 0.1% 1SD (n=4)). No material was obtained in the <30 kDa fraction of the method 2 extracts.

**Figure 5.**
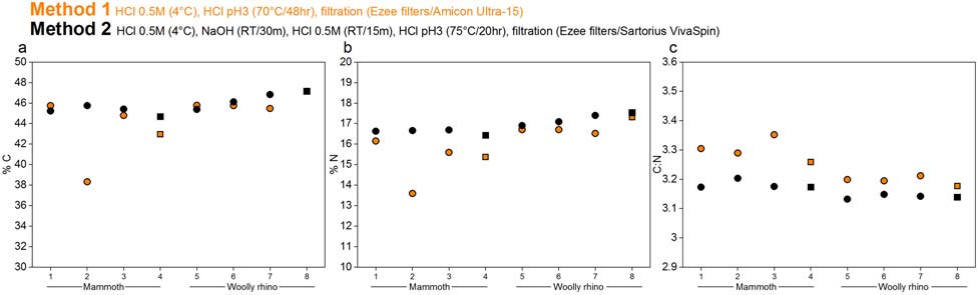
%C, %N, and C:N ratios of collagen extracted from the mammoth (samples 1-4) and woolly rhino (samples 5-8) bones pretreated with Method 1 (orange) and Method 2 (black). Squares are whole bone samples, and circles are bone powder samples.

The %C, %N and C:N values of all extracts pretreated with the two methods fall within the accepted ranges of well-preserved collagen (C:N = 2.9-3.6; %C = 30-46%; %N = 10-17%) (van Klinken 1999) (Figure 5 and Supplementary Table S1). The C:N ratios of the Method 1 >30 kDa extracts (3.25 ± 0.06 1SD) are slightly higher than the Method 1 <30 kDa extracts (3.14 ± 0.02 1SD, paired t-test *p* value = *p*-value = 0.0002977, n=7). These, in turn, are actually more similar to the Method 2 >30 kDa extracts (3.16 ± 0.02 1SD). The slightly higher C:N ratios from Method 1 compared to Method 2 (paired t-test *p* value = 0.0001255, n=8) is similar to the pattern observed in a larger dataset reported in Colleter et al. 2019, although that study used the two methods on bones from different individuals (Method 1 n=99: 3.33 ± 0.11 1SD; Method 2 n=18: 3.23 ± 0.11 1SD). van Klinken (1999) reported that modern bones had an average C:N of 3.29 ± 0.27 based on >2000 samples. More recently, Guiry and Szpak (2020) reported a C:N value of 3.23 ± 0.04 from amino acid compositions of modern mammal bones (n=24), and recommended that isotopic compositions of modern mammal/bird bones only be considered reliable when their C:N values fall within 3.00-3.28. Based on these criteria, the mammoth and woolly rhino bones from Methods 1 and 2 would both be considered perfectly suitable for ^14^C dating and for dietary isotope studies. Although the Method 1 >30 kDa extracts are, in fact, closer to the mean of the modern bone C:N values, the slightly higher C:N values may reflect a low-level presence of C-rich humic acids retained in this fraction compared to the Method 1 <30 kDa fraction (humic acids would not have passed through the ultrafilter) and the Method 2 >30 kDa fraction (humics removed by the NaOH step). Guiry and Szpak (2020) reported significant shifts to more negative δ^13^C values with differences in C:N of just 0.03. We did observe significantly lower δ^13^C values (0.14 ‰) for Method 1 than Method 2 (paired t test *p*-value = 0.01107) associated to higher C:N of 0.1 for Method 1, but this trend was not considered significant since all the stable isotopic compositions were within instrumental error. This indicates that any humic acids present in the Method 1 >30 kDa extracts were sufficiently high to affect the ^14^C dates due to the extreme age of the two bones but were not present in sufficient quantities to affect the stable isotopic compositions.

The %C and %N values of mammoth sample 2 (palaeodiet) are lower than the other replicates pretreated with the same criteria, perhaps due to variation of bone preservation at different sampling locations but are still well within accepted ranges.

**Figure 6.**
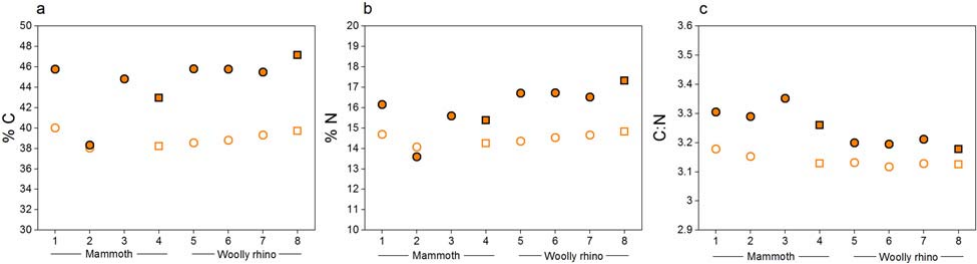
%C, %N, and C:N ratios of collagen extracted from the mammoth (samples 1-4) and woolly rhino (samples 5-8) bones pretreated with Method 1. Full orange points are data from >30 kDa fraction and the unfilled points are the <30 kDa fraction. Squares are whole bone samples, and circles are bone powder samples.

Only the Method 1 extracts produced enough material in the <30 kDa fraction for analysis with EA-IRMS. Generally, the %C, %N and C:N were lower for the small molecular weight fraction compared to the higher molecular weight fraction (Figure 6). The only exception was the Mammoth sample 2 (as already noted above). It is likely that the longer duration and higher temperature of the gelatinisation step used in Method 1 lead to increased hydrolysation of collagen (Semal and Orban 1995; Beaumont et al. 2010; Fewlass et al. 2019), which resulted in a higher amount of low molecular weight material passing through the ultrafilter with significantly lower %C compared to the >30 kDa fraction (median difference: 6.2%; Wilcoxon signed rank test: V = 28, *p*-value = 0.01563).

#### 3.1.3. ^14^C results

**Figure 7.**
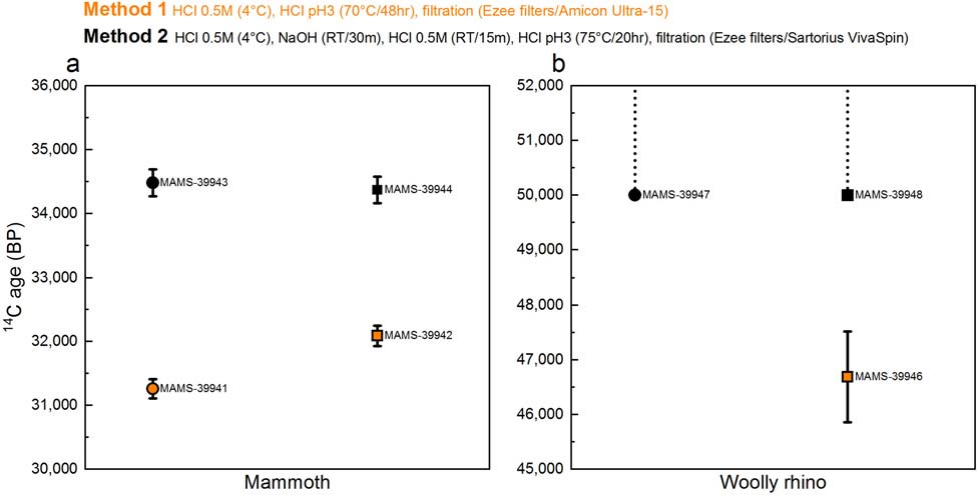
Comparison of radiocarbon dates from mammoth (a) and woolly rhino (b) collagen extracts pretreated with the Method 1 (orange) Method 2 (black) extraction protocols. Squares are whole bone samples and circles are bone powder samples. Error bars are 1σ.

The ^14^C dates obtained from the mammoth and woolly rhino collagen extracted using Method 2 (dating protocol) are in keeping with previous dates obtained from these bones, from powdered and whole bone pieces (Fewlass et al. 2019). In contrast, the ^14^C dates obtained from collagen extracted using Method 1 (Palaeodiet protocol) are younger for both bones (Figure 7 and Supplementary Table S1), indicating that the Method 1 extracts still contained a modern C contribution following pretreatment. As the Method 1 and 2 extracts were graphitised and measured in the AMS at the same time, it is unlikely that this contamination occurred only for Method 1 extracts during graphitisation or measurement.

Two factors in the pretreatment protocols are the most likely candidates for causing these age discrepancies. Firstly, the inclusion of the NaOH step in Method 2 to remove humic acid contamination. Talamo and Richards (2011) reported similarly young ages from the same bones using extraction protocols that also excluded the NaOH step. We observed a distinct colour change (from clear to yellow) during the NaOH wash during the Method 2 pretreatment for both bones. We, therefore, consider this the most likely cause for the under-estimated ages, as previously demonstrated in DeNiro and Epstein (1981), Ambrose (1990), Szpak et al., (2017a), Jørkov et al., (2007) and Guiry and Szpak (2020). Alternatively, different brands of ultrafilter and filter cleaning protocols were used in the two methods. It could be that the cleaning protocol used did not sufficiently clean the Amicon-15 filter, but this hypothesis requires further testing.

Overall, the results from Experiment A indicate that both collagen extraction protocols are suitable for palaeodietary studies. The ^14^C protocol (Method 2) yields a higher amount of collagen than the palaeodietary protocol (Method 1). Whilst the Method 1 gelatinisation step takes longer (48 h compared to 20 h), Method 2 is more labour-intensive due to the additional NaOH/HCl steps. However, the most important insight was that Method 1 did not produce accurate ^14^C dates, likely due to the omission of the NaOH step, indicating that this method is unsuitable for ^14^C dating. The indistinguishable isotopic compositions between the methods indicates that any humic acid contaminants remaining in the Method 1 extracts were sufficiently low in quantity not to impact the stable isotopic values. Yet given the very old ages of both bones (≥35 ka), even very low quantities of exogenous carbon would have significant impacts on the ^14^C dates. As a caveat, this test is based on only two bone samples with unknown levels of humic acid contamination. For bones without humic acid contaminants, Method 1 may well yield accurate results. As it is not possible to determine the level of humic acid contamination in advance, the Method 2 protocol should be used for all bone samples to be dated.

### 3.2. Experiment B

#### 3.2.1. Collagen yield

**Figure 8.**
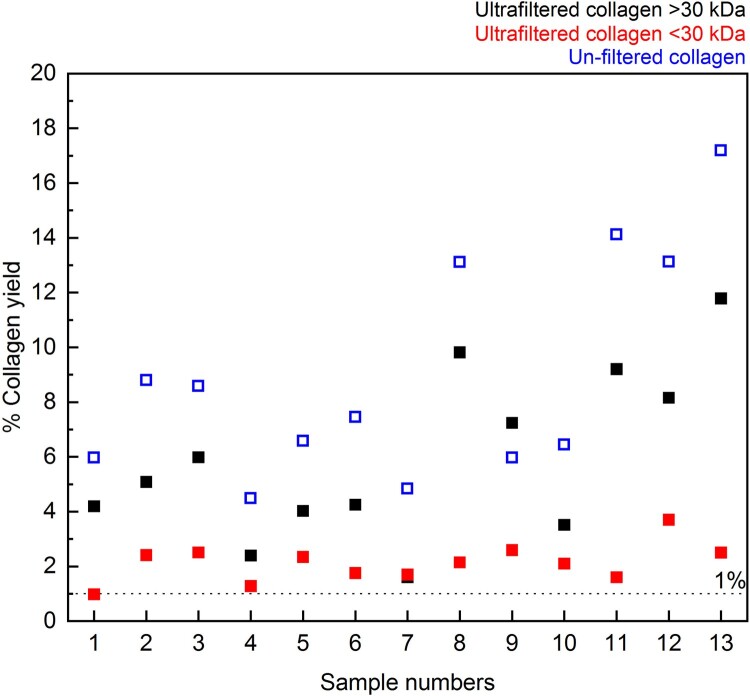
Differences between % collagen yield for ultrafiltered collagen (>30 kDa in black and <30 kDa in red) and non-ultrafiltered collagen (blue) extracted from 13 bone samples in Experiment B.

As observed in previous tests (Jørkov, Heinemeier, and Lynnerup 2007; Hüls, Grootes, and Nadeau 2009; Brock et al. 2013; Szpak, Krippner, and Richards 2017a), the collagen yield was reduced by the inclusion of the ultrafiltration step (Figure 8 and Supplementary Table S2). However, the observation that collagen yields are lower in this study may be somewhat biased as the sample set was selected specifically for the high <30 kDa yield to gain enough material for this fraction to be dated. In many cases, there is little or no material after freeze-drying of the <30 kDa fraction (for example, from the bones pretreated with Method 2 in Experiment A). In those cases, the inclusion of the ultrafiltration step may not have as dramatic an impact on the yield as those observed here.

It is interesting to note that the >30 kDa and <30 kDa collagen yields do not always add up to the collagen yield of the extracts without ultrafiltration, implying that a small amount of collagen may be lost elsewhere between the two methods. As both methods include the same conditions for the NaOH and gelatinisation stages, it could be that a small amount is also lost during filtering with the Ezee filter (not used in Method 3) or is retained on the ultrafilter itself.

#### 3.2.2. Elemental and stable isotopic values

**Figure 9.**
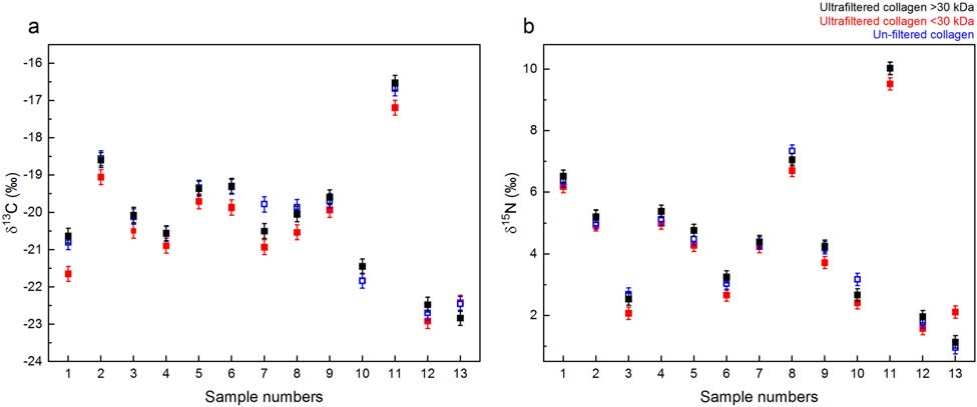
δ^13^C and δ^15^N values of collagen extracts: >30 kDa fraction (black), <30 kDa fraction (red) and Method 3 extracts without ultrafiltration (blue). The error bars show an analytical error of 0.2‰ (1σ) based on repeated analysis of both internal and international standards.

The stable isotopic values (δ^13^C and δ^15^N) do not differ between the Method 2 >30 kDa and <30 kDa fractions, and the Method 3 non-ultrafiltered collagen extracts (Figure 9 and Supplementary Table S2). This indicates that the inclusion of the ultrafiltration step did not affect the stable isotopic values, in agreement with the findings of Cersoy et al. (2017). Although, the δ^13^C of Method 2 <30 kDa extracts appears systematically lower than those of the Method 2 >30kD extracts, paired t-tests between the δ^13^C of the Method 2 <30 kDa with Method 2 >30 kDa and Method 3 do not reveal a *p* value below 0.05, suggesting that the offset is not significant.

**Figure 10.**
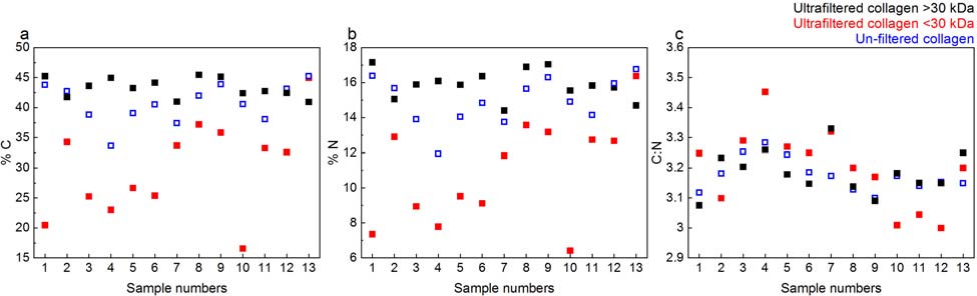
%C, %N, and C:N ratios of collagen extracts in Experiment B: >30 kDa fraction (black), <30 kDa fraction (red) and Method 3 extracts without ultrafiltration (blue). Instrumental error is ± 0.2%.

Overall, the %C and %N values are generally highest for the Method 2 >30 kDa fraction (falling within accepted ranges) and much lower for the <30 kDa fraction (in some cases, below accepted ranges). The %C and %N values of the Method 3 extracts (no ultrafiltration) of the same bone are roughly equal or slightly lower than the >30 kDa ultrafiltered fractions. The C:N values of the >30 kDa (>30 kDa mean: 3.18± 0.07 1SD) and unfiltered Method 3 extracts (Method 3 mean: 3.18± 0.05 1SD) are roughly equal but the standard deviation of the C:N values of the <30 kDa fraction is slightly higher (<30 kDa mean: 3.2 ± 0.13 1SD). However, the C:N values of all fractions fall within the accepted range of 2.9-3.6, and even within the 3.00-3.28 range of modern mammal bones reported by Guiry and Szpak (2020) (Figure 10 and Supplementary Table S2).

As the %C and %N of the <30 kDa fraction fall below the accepted ranges for well-preserved collagen whilst the C:N ratio is still “acceptable”, this re-confirms that it is necessary to consider %C and %N values just as carefully as the derived C:N ratio (Ambrose 1990; Szpak, Metcalfe, and Macdonald 2017b). All should be reported as quality indicators in publications, rather than just the commonly reported C:N value.

#### 3.2.3. ^14^C results

**Figure 11.**
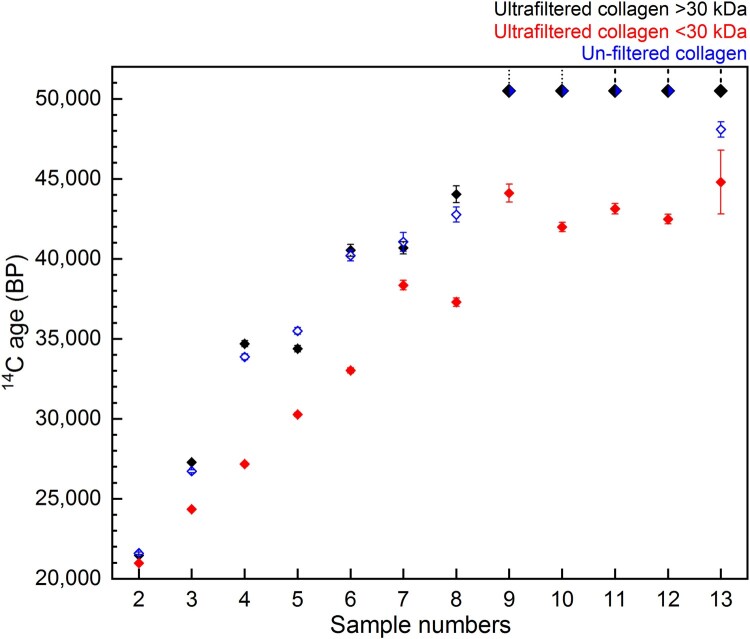
Comparison of ^14^C ages ± 1 SD error from the bones in Experiment B dating >20,000 BP: >30 kDa fraction (black) and <30 kDa fraction (red) of ultrafiltered collagen extracted using Method 2, and collagen extracted using Method 3 without ultrafiltration (blue). Samples dating to >50,000 BP are indicated by the dashed line.

Supplementary Table S2 and Figure 11 show the ^14^C ages obtained from the Method 2 ultrafiltered collagen fractions (>30 kDa and <30 kDa) and the collagen extracted using Method 3 (excluding ultrafiltration).

For nine bones out of the 13, there was no difference in ^14^C age between the >30 kDa ultrafiltered extract and Method 3 extract (without ultrafiltration) (Figure 11). This demonstrates again that in some instances modified versions of the Longin protocol without additional ultrafiltration steps are suitable pretreatment methods to remove exogenous carbon and obtain accurate ^14^C results (as demonstrated by multiple Method 3 extracts dating >50,000 BP). In three cases (∼23%), the dates from the unfiltered Method 3 extracts were younger than the >30 kDa ultrafiltered extracts from the same bone (outside 2σ). This indicates that the ultrafilters removed some contaminants that remained in Method 3 extracts, resulting in a more accurate (older) age. In one of these cases, the age of the bone (sample 13, Figure 11) is >50,000 BP, representing the very limit of the method. In one ∼35,000 year old bone (sample 5, Figure 11), the date from the Method 3 extract was older than the >30 kDa extract (just outside the 2σ range). It is not known why one bone yielded an older age using Method 3. It is possible that the corresponding >30 kDa extract was contaminated during graphitisation or AMS measurement, or the membrane of the ultrafilter used for this sample was not cleaned sufficiently prior to use.

Most strikingly, in all 13 samples the <30 kDa fraction was much younger than both the >30 kDa fraction and Method 3 extract from the same bone (this was also observed by Hüls et al. 2017; 2009). The Milli-Q water from the cleaning steps of the ultrafilters is regularly measured with an elemental analyser (on chromosorb) to monitor carbon content. These measurements indicate that any carbon remaining on the filter after the cleaning steps is below the level of detection in the EA. Hüls et al. (2009) also reported that although C measurements of water following cleaning suggested almost complete glycerin removal, scanning electron microscopy still showed residue remaining on the filters after cleaning, although their study used different ultrafilters and cleaning protocols. The >50,000 BP ^14^C dates from the >30 kDa fractions demonstrate that the ultrafilter is not introducing modern C to the large molecular weight fraction in the top of the ultrafilter, but the young ages of the <30 kDa fraction clearly indicate contamination of the small molecular weight fraction as it passes through the filter.

Although the <30 kDa fractions have lower and more variable %C and %N values, there is no difference in the δ^13^C and δ^15^N values compared to the other fractions. Some labs retain this fraction for palaeodietary analysis if sufficient material is lacking in the >30 kDa fraction (Sealy et al. 2014). Our results demonstrate that this material, having passed through the filter, should not be used for ^14^C dating under any circumstances as it will not provide accurate results. Further testing with other filters is necessary to determine if this is the case only for the Sartorius Vivaspin Turbo15 ultrafilters or other brands.

#### 3.2.4. FTIR analysis of extracted collagen

**Figure 12.**
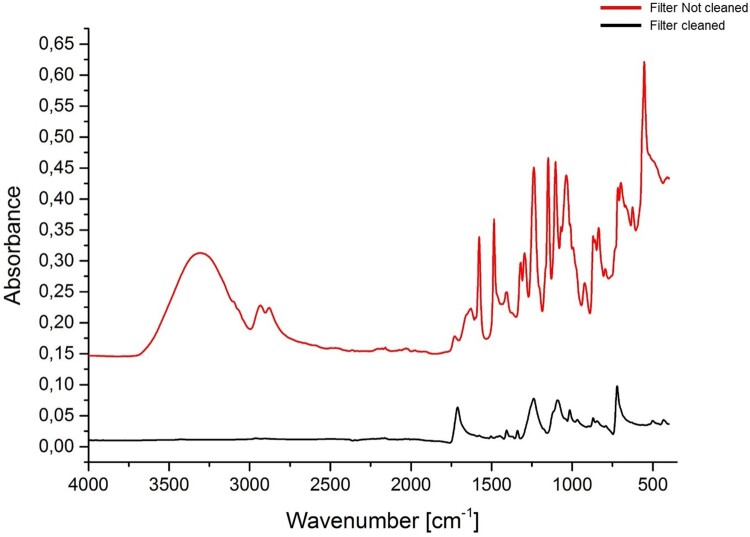
FTIR spectra of the Sartorius Vivaspin Turbo 15 ultrafilter membrane used in the Method 2 prior to cleaning (red) and after cleaning (black).

Since the ultrafilter membrane is coated with glycine to maintain flexibility, we performed FTIR analyses on the filter membrane before and after washing (Figure 12). The uncleaned membrane filter presented peaks in the same regions as collagen, which could interfere with the analysis of collagen extracts. However, after the cleaning procedure, the majority of the bands disappeared.

We compared FTIR spectra of the >30 kDa collagen fraction, <30 kDa collagen fraction and the Method 3 extract from each bone in Experiment B to see if we could observe or identify the contamination in the <30 kDa fraction causing the younger ^14^C ages. We observed no difference between the FTIR spectra of the Method 3 extracts, >30 kDa and <30 kDa fractions for any of the bones, except R-EVA 616 where little <30 kDa collagen was present. The FTIR spectra of the ultrafiltered collagen extracts (>30 kDa and <30 kDa fractions) were also compared to FTIR spectra of an unclean/cleaned ultrafilter membrane, but no correlating peaks were identified. As FTIR analysis detects the composition of the material regardless of the quantity, in theory even low levels of contamination should be visible in the spectra as additional peaks. However, if the contamination leading to the young ages of the <30 kDa collagen is the result of passing through the ultrafilter membrane, as seems most parsimonious (rather than resulting from contamination during graphitisation/measurement), this indicates that the FTIR does not identify contaminants present in very low quantities. Alternatively, it may be the case that peaks from the contaminant overlap with the collagen peaks and are therefore masked.

### 3.3. Elemental data as a quality indicator

The Experiment B <30 kDa extracts, as well as the Method 1 >30 kDa extracts (in Experiment A) had elemental values falling in the range of modern collagen extracts (Figures 5 and 10), but the ^14^C dates were erroneously young (Figure 7). This demonstrates that exogenous carbon was present in the extracts without this being apparent in the quality criteria. This possibility was discussed by van Klinken (1999) and demonstrates that multiple quality indicators should be considered for each extract.

In some cases, contamination causing high C:N ratios can be the result of material endogenous to the bone or exogenous material that is contemporaneous to the dated material. In these cases, ^14^C ages of contaminated material will not necessarily be inaccurate and the limit imposed by van Klinken (1999) should be taken as a simple warning. We note and report here two cases (Table 2) where bones with C:N ratios outside of accepted ranges produced ^14^C dates in keeping with expectations and historical data.

**Table 2.** Radiocarbon ages, chemical indicators including the collagen yield, %N, %C, C:N and stable isotopic values (δ^13^C and δ^15^N), and FTIR analysis of Louise de Quengo (R-EVA 3201) and Mezzena mandible (R-EVA 1395). The extracts were also analysed at the MPI-EVA in Leipzig, Germany (see section 2.6 for methods).

Sample	Origin	collagen yld (mg)	collagen yld (%)	δ^13^C (‰)	δ^15^N (‰)	%C	%N	C:N	AMS Nr.	^14^C age ± 1σ err (BP)	FTIR Comment
R-EVA 3201	France	177.3	38.9	−22.6	14.4	52.1	12.4	4.9	MAMS-30094	321 ± 14	Collagen peaks and higher intensity of the 2900 cm^−1^ band
R-EVA 1395	Italy	5.9	1.3	−21.8	7.1	8.2	2.4	4.1	MAMS-24343	5580 ± 26	Collagen peaks and higher intensity of the 2900 cm^−1^ band

R-EVA 3201 is a bone belonging to Louise de Quengo, a natural mummy discovered in 2014 in a lead coffin in Rennes, Brittany, dating from the seventeenth century AD. Her birth and death dates are known (1584-1656 AD) (Le Cloirec 2016). Colleter et al. (2019) previously extracted collagen to investigate her diet using the Method 2 protocol. The bone collagen (R-EVA 3201) had a high C:N ratio (4.9), but a peak in the FTIR spectra around 2900 cm^−1^, the high % of collagen and %C, as well as a low δ^13^C value all suggested this was due to the presence of endogenous lipids in the >30 kDa fraction (Table 2). The presence of coeval exogenous material is considered highly unlikely as the body was buried in a hermetic lead coffin and the bone sample (collected during the autopsy of the mummy) was cleaned of all tissues and organic matter before analyses. Although the sample was deemed unsuitable for dietary interpretation, the bone collagen was AMS dated to 1500–1640 cal AD (2σ range calibrated with IntCal20 (Reimer et al. 2020) using OxCal 4.4 (Bronk Ramsey 2009)), close of the death date of Louise de Quengo (10th March AD 1656), considering bone turnover rates of several decades. In spite of the elevated C:N ratio, the ^14^C date, therefore, appears accurate, further indicating that the contaminant was endogenous to the individual (Guiry, Szpak, and Richards 2016).

R-EVA 1395 is a mandible fragment originally thought to belong to a Neanderthal (previously described in Talamo et al. (2016b), reported as S-EVA 32612). This sample was stored at the Museum of Natural Science in Verona (Italy) together with other pieces of human skull from the same layer attributed to the same individual. We previously dated two of the associated cranial bone fragments, which resulted in ^14^C ages of ∼5500 cal BP, with collagen yields >1% and C:N values within accepted ranges (3.3-3.4) (Talamo et al. 2016b). A high C:N value (4.1) was obtained from the R-EVA 1395 mandible fragment, which only yielded 1.3% collagen (close to the 1% minimum requirement). The %C of collagen extracted was very low (8.2%), but this was attributed to the presence of inorganic substances in the extract rather than contamination by carbon from modern sources (van Klinken 1999). Despite the high C:N value, the date obtained from the mandible (MAMS-24343: 5580 ± 26 ^14^C BP) agreed fully with the dates from the associated cranial fragments, indicating that it was not significantly affected by modern carbon contamination (Table 2). Although originally considered Neanderthal, all the ages indicate a Neolithic origin for this individual.

These rare instances indicate that in certain circumstances it may be possible to obtain accurate ^14^C dates from collagen extracts with C:N values falling outside the “accepted” ranges. This should only be attempted if sufficient evidence is available for a thorough understanding of the origin of potential contaminants (i.e. endogenous lipids will not necessarily produce inaccurate ages). For samples of high value (e.g. human remains or bone/tooth/ivory artefacts) where it is possible to confirm the age through independent correlation (e.g. historical information, dates of other high-quality material from the same context/layer) it can be worth attempting ^14^C dating.

### 3.4. Stable isotopic data from different extraction protocols

The offset between the δ^13^C obtained with the Methods 1 and 2 or 2 and 3 (Δ^13^C _1-2,_ Δ^13^C _1-3,_ Δ^13^C _2-3_) do not correlate with the δ^15^N offsets. No correlation of these offsets was observed with the ΔC:N. These absences of correlation argue for the absence of impact of the chosen extraction protocol on the δ^13^C and δ^15^N compositions. Despite the differences in collagen yield and ^14^C ages, all three protocols are therefore appropriate methods to extract collagen for palaeodietary studies. Therefore, in contrast to a few studies where differences in δ^13^C values were observed for collagen extracted with different protocols (Jørkov, Heinemeier, and Lynnerup 2007; Szpak, Krippner, and Richards 2017a), we observed consistency in stable isotopic values between the three protocols, as had been noted in several studies (Chisholm et al. 1983; Pestle 2010; Caputo et al. 2012; Sealy et al. 2014; Cersoy et al. 2017). However, we should point out that the tested protocols included ultrafiltration and/or NaOH steps to remove potential contaminants, which may affect the carbon and nitrogen isotope ratios. In contrast to the studies by Jørkov et al (2007) and Szpak et al., (2017a), we did not use samples known to be heavily contaminated with humic acid or lipids, which may explain the differences observed in our findings.

## 4. Conclusions

Comparing the collagen extraction protocol in use at the MPI-EVA for stable isotopic analysis (Method 1; modified Richards and Hedges 1999) and the method in use since 2011 for radiocarbon dating (Method 2; Talamo and Richards 2011; Fewlass et al. 2019), we observed a substantial improvement in recovering collagen, which likely results from the different conditions of the gelatinisation step. The inclusion of the NaOH step in Method 2 did not negatively impact the collagen yield in this study. This experiment again indicates that the pretreatment of whole bone fragments yields a higher amount of collagen compared to pretreating bone powder, although the demineralisation stage is more time consuming for whole bone pieces. The stable isotopic values did not differ between the extracts from the different methods, indicating both are suitable for palaeodietary analysis. However, the collagen extracts dated using the palaeodiet Method 1 were younger than the ^14^C dates obtained with Method 2. This is most likely due to the omission of the NaOH step in Method 1, with humic acid contaminants responsible for the inaccurate ^14^C results. The results, therefore, indicate that Method 1 is not suitable for extracting collagen for ^14^C dating. It is worth remembering that this test is based on only two bones and may vary in other cases where collagen preservation is lower or the level of humic acid contamination varies.

Experiment B demonstrated again that in many cases an acid–base-acid collagen extraction protocol is sufficient to produce reliable ^14^C dates from Palaeolithic bones, even at the very limit of the method and for bones of “background” age (>50,000 BP). Although the inclusion of the ultrafiltration step can decrease the collagen yield (although samples used here were specifically selected for their high <30 kDa yield), in some cases it leads to older ^14^C dates. This likely depends on the conditions of the burial environment and the level of degradation/contamination of each sample, which is difficult to determine in advance. For this reason and especially for Palaeolithic samples, we still consider it useful to include the ultrafiltration step. The results show that the filter did not contaminate the >30 kDa fraction (selected for dating), but significant contamination was added to the small molecular weight fraction passing through the filter, rendering it unsuitable for ^14^C dating.

In all cases, the stable isotopic values did not vary substantially between collagen pretreated with different protocols, indicating that all were suitable methods to use in palaeodietary studies. Although the quality criteria used here are important quality indicators, clearly they are not infallible. In both Experiment A and Experiment B, collagen extracts with elemental values (%C, %N, C:N) within established ranges of well-preserved collagen produced erroneous ^14^C dates, and contaminants from the ultrafilter were not observable in the FTIR spectra of the <30 kDa fraction. In conclusion, in order to be confident of ^14^C data, it is necessary to consider a range of quality indicators for each collagen extract, rather than rely on only one.

## Supplementary Material

Supplemental Material
