# Supplementary material for: “Here we go again”: the inspection of collagen extraction protocols for 14C dating and palaeodietary analysis
Source: Sci Technol Archaeol Res. 2021 Jul 20;7(1):62–77. doi: 10.1080/20548923.2021.1944479 (PMC8300532; doi:10.1080/20548923.2021.1944479)
Supplement: Supplemental Material [file YSTA_A_1944479_SM2195.docx]

**Supplementary Information**

**Details on carbon and nitrogen isotope analyses**

The standard used for calibration was the methionine MET (Szpak et al. 2017). The full uncertainty estimated from Szpak et al. (2017) are of 0.34 ‰ for carbon isotopes and 0.26 ‰ for nitrogen isotopes. The quite high carbon isotope uncertainty is due to our systematical measurement of the sample CH7 (long term value:-31.8 ‰) which is always about 0.5 ‰ lower than the published value.

**References**

Rey, L., Domingo C. Salazar-García, F. Santos, S. Rottier, et G. Goude. « A multi-isotope analysis of Neolithic human groups in the Yonne valley, Northern France: insights into dietary patterns and social structure ». *Archaeological and Anthropological Sciences* 11, n^o^ 10 (2019): 5591‑5616.

Szpak, Paul, Jessica Z. Metcalfe, et Rebecca A. Macdonald. « Best Practices for Calibrating and Reporting Stable Isotope Measurements in Archaeology ». *Journal of Archaeological Science: Reports* 13 (1 juin 2017): 609‑16. <https://doi.org/10.1016/j.jasrep.2017.05.007>.
